# NMR Structure and Dynamics of TonB Investigated by Scar-Less Segmental Isotopic Labeling Using a Salt-Inducible Split Intein

**DOI:** 10.3389/fchem.2020.00136

**Published:** 2020-03-19

**Authors:** Annika Ciragan, Sofia M. Backlund, Kornelia M. Mikula, Hannes M. Beyer, O. H. Samuli Ollila, Hideo Iwaï

**Affiliations:** Institute of Biotechnology, University of Helsinki, Helsinki, Finland

**Keywords:** inteins, protein ligation, NMR spectroscopy, segmental isotopic labeling, TonB, intrinsically disordered protein, multi-domain proteins, protein dynamics

## Abstract

The growing understanding of partially unfolded proteins increasingly points to their biological relevance in allosteric regulation, complex formation, and protein design. However, the structural characterization of disordered proteins remains challenging. NMR methods can access both the dynamics and structures of such proteins, yet suffering from a high degeneracy of NMR signals. Here, we overcame this bottleneck utilizing a salt-inducible split intein to produce segmentally isotope-labeled samples with the native sequence, including the ligation junction. With this technique, we investigated the NMR structure and conformational dynamics of TonB from *Helicobacter pylori* in the presence of a proline-rich low complexity region. Spin relaxation experiments suggest that the several nano-second time scale dynamics of the C-terminal domain (CTD) is almost independent of the faster pico-to-nanosecond dynamics of the low complexity central region (LCCR). Our results demonstrate the utility of segmental isotopic labeling for proteins with heterogenous dynamics such as TonB and could advance NMR studies of other partially unfolded proteins.

## Introduction

Segmental isotopic labeling facilitates nuclear magnetic resonance (NMR) studies of large or multi-domain proteins, and proteins with low complexity regions by alleviating NMR signal overlaps (Yamazaki et al., 1998; Xu et al., 1999; Skrisovska and Allain, 2008; Volkmann and Iwaï, 2010). Segmentally introducing isotopic labels into proteins also allows their NMR spectroscopic investigation by restricting the emerge of signals to a particular protein domain or region in the full-length protein context (Busche et al., 2009; Buchinger et al., 2010; Minato et al., 2012, 2017; Shiraishi et al., 2018; Wiegand et al., 2018). Several methods, including expressed protein ligation (EPL), protein-*trans* splicing (PTS), fragment ligation using sortases or asparagine endopeptidases have been used for segmental isotopic labeling (Skrisovska and Allain, 2008; Muona et al., 2010; Freiburger et al., 2015; Kwon et al., 2015; Williams et al., 2016; Frederick et al., 2017; Mikula et al., 2017, 2018; Wiegand et al., 2018). Albeit these various available methods, segmental isotopic labeling for structural studies remains challenging due to the requirement of relatively high quantities (>mg scale) and high purity. Moreover, segmentally isotopic-labeled samples prepared by these methods often contain mutations at the ligation site due to the technical requirements. For example, sortase-mediated ligation requires a sequence of “LPXTGG,” remaining as a “scar” in the ligated product (Freiburger et al., 2015; Williams et al., 2016). PTS by the widely used split DnaE intein from *Nostoc punctiforme (Npu*DnaE) and EPL require a cysteine mutation (remaining as a “scar” in the product) at the ligation site. Introducing such modifications could potentially influence the structure and dynamics of the proteins of interest. The “scar” problem at the ligation site stimulated several protein engineering attempts of inteins, such as converting the nucleophilic cysteine at the so-called +1 position to serine (Lockless and Muir, 2009; Cheriyan et al., 2013). The recent development of the split MCM2 intein from *Halorhabdus utahensis (Hut*MCM2) could alleviate the “scar” problem as well as the solubility issue associated with split inteins (Ciragan et al., 2016). The minimal sequence requirement for PTS using the split *Hut*MCM2 intein is theoretically a Ser at the +1 position, which is more prevalent than cysteine among diverse proteins (Ciragan et al., 2016). Additionally, the halophilic intein is highly soluble even after artificial splitting into *trans*-reacting fragments, making this intein very attractive for scar-less segmental isotopic labeling (Aranko et al., 2014a,b; Ciragan et al., 2016).

**Figure 1:**
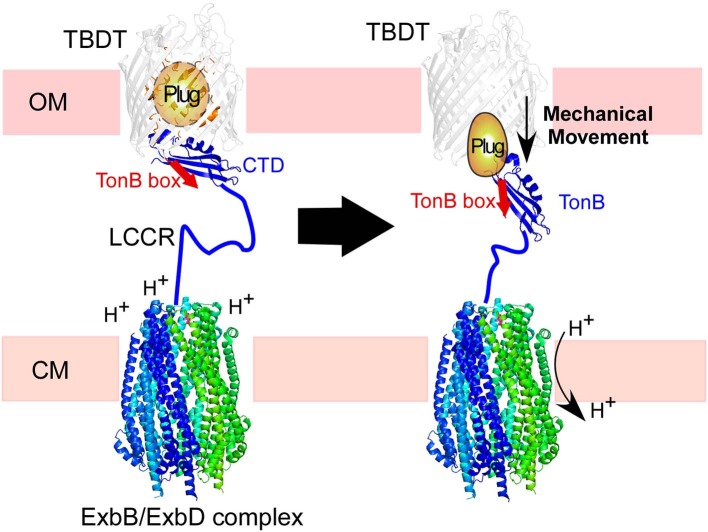
Illustration of TonB-dependent transporters (TBDT) in the cell envelope of Gram-negative bacteria. The C-terminal domain (CTD) of TonB is proposed to interact with the TonB box in the plug domain of TBDT (left). The proton-motive force across the cytoplasmic membrane (CM) is transduced by the ExbB/ExbD complex to induce mechanical force via TonB. The low complexity central region (LCCR) spans in the periplasmic space to interact with TBDTs in the outer-membrane.

**Figure 2:**
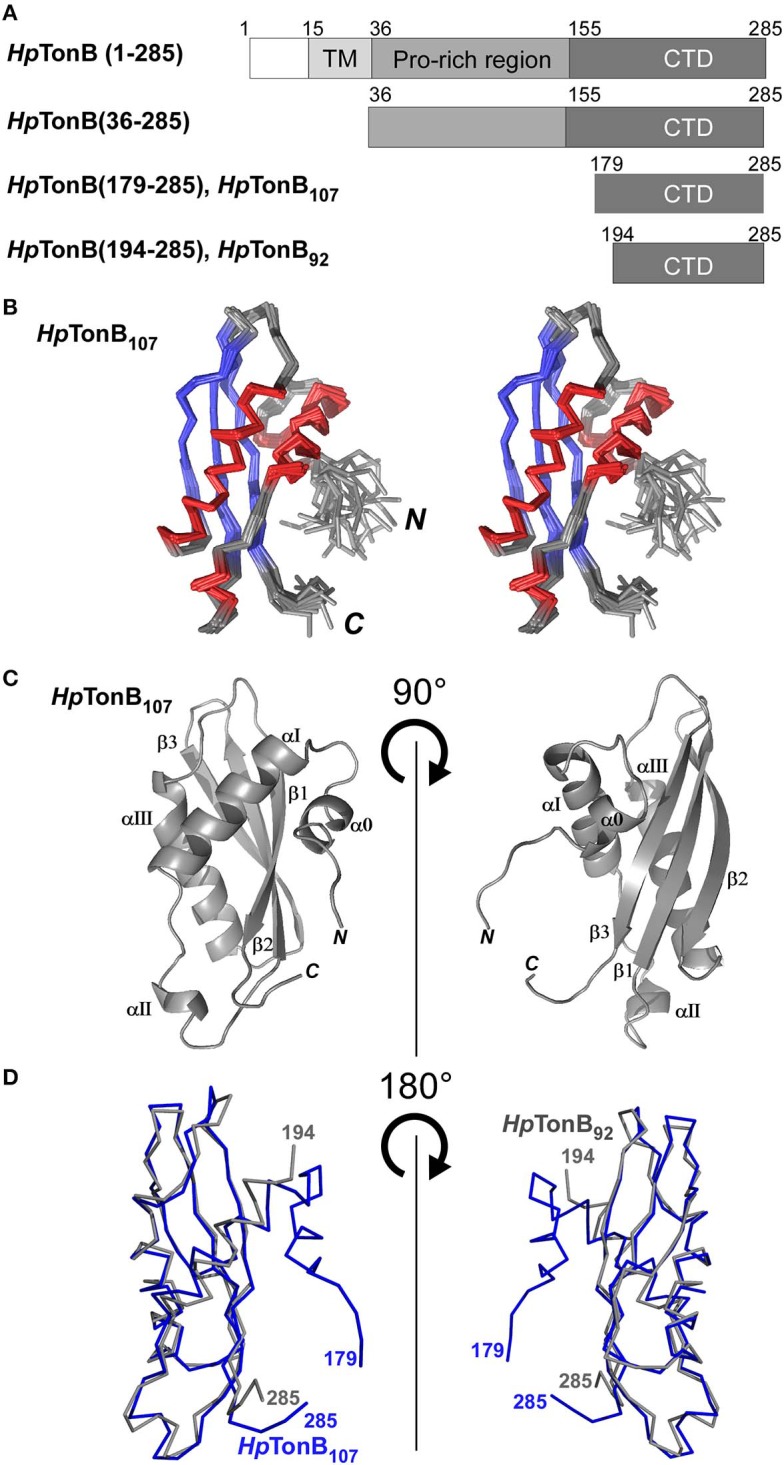
NMR solution structure of *Hp*TonB_107_ **(A)** Schematic representations of the domain organization of the full-length TonB and dissected *Hp*TonB variants used in this study. **(B)** A stereo-view of an ensemble of the 20 NMR-refined conformers of *Hp*TonB_107_. The regions for α-helices and β-sheets are colored in red and blue, respectively. **(C)** Cartoon drawings of the *Hp*TonB_107_ structure with the secondary structure elements labeled. **(D)** A superposition of the two NMR structures of *Hp*TonB_107_ (in blue) and *Hp*TonB_92_ (in gray). The residue numbers for the termini are shown.

Many multi-domain proteins or regions lack a well-structured three-dimensional fold. These intrinsically disordered proteins (IDPs) and intrinsically disordered protein regions (IDRs) are very prevalent, and their importance has been increasingly recognized in allosteric regulation, complex formation, and protein design (Chen et al., 2013; Papaleo et al., 2016; Berlow et al., 2018; Keul et al., 2018). However, IDRs typically have low complexity sequences, and their NMR signals tend to degenerate due to the lack of a well-defined fold. The higher degeneracy of IDRs compared to highly structured proteins could hinder their detailed NMR analysis, calling for strategies to alleviate signal overlaps when studying IDPs and IDRs (Nabeshima et al., 2014; Frederick et al., 2017). We chose TonB from Gram-negative bacteria as an example of multi-domain proteins where a central proline-rich low complexity region (LCCR) connects a globular C-terminal domain (CTD) to an N-terminal transmembrane (TM) domain (Figures 1, 2A, Figure S1). TonB plays an essential role in the TonB-dependent transport mechanism transducing the cytoplasmic proton motive force (PMF) to the TonB-dependent transporters (TBDT) in the outer membrane (OM) (Figure 1) (Postle and Larsen, 2007). The transmembrane domain of TonB is associated with the inner membrane-bound proteins ExbB and ExdD, and it interacts with TBDTs in the outer-membrane via a globular C-terminal domain (CTD) (Figure 1). The LCCR likely spans across the periplasm for positioning the CTD to contact TBDTs in the outer membrane (Shultis et al., 2006). The functional role of the LCCR in the periplasmic space is poorly understood, although an extended polyproline type II helical conformation has been proposed (Evans et al., 1986; Brewer et al., 1990; Köhler et al., 2010). The LCCR connecting N-terminus and CTD is highly divergent but contains a proline-rich repetitive sequence in nearly every case, presumably having high degeneracy of NMR signals. Thus, the LCCR could hinder the detailed analysis of the full-length TonB by NMR (Figure S1).

Here, we report the NMR structures of the newly dissected CTD of TonB from *Helicobacter pylori (Hp*TonB) and analyzed the effects of the LCCR on the conformational dynamics of TonB by comparison with two differently dissected CTDs. We used a scar-less segmental isotopic labeling approach utilizing a salt-inducible split intein to alleviate the high degeneracy of NMR signals due to the proline-rich low complexity region of TonB.

## Results

### NMR Solution Structure of TonB (179–285) From *Helicobacter pylori* (*Hp*TonB_107_)

First, we determined the NMR solution structure of a 107-residue CTD of *Hp*TonB (179–285) (*Hp*TonB_107_) for the comparison with *Hp*TonB_92_ which has been previously reported (Ciragan et al., 2016; Figure 2, Figure S2, Table 1). Three-dimensional structures of variously dissected CTDs of TonB from several Gram-negative bacteria determined by NMR and X-ray crystallography demonstrated variations mainly in the N- and C-terminal regions, suggesting that the dissection positions for the CTD could influence the conformation of the CTD (Chang et al., 2001; Ködding et al., 2005; Peacock et al., 2005; Chu et al., 2010; Oeemig et al., 2018). Notably, the previously determined *Hp*TonB_92_ indicated notable chemical shift differences over the entire primary structure compared with the chemical shifts of *Hp*TonB (36–285), suggesting possible structural differences of the CTD in the full-length context (Ciragan et al., 2016). The newly determined NMR structure of *Hp*TonB_107_ with a slightly longer length is very similar to the previously determined NMR structure of *Hp*TonB_92_ with the root-mean-square deviation (RMSD) of 1.37 Å for the backbone heavy atoms for residues 195–282 (Figures 2A,D). *Hp*TonB_107_ consists of one β-sheet composed of three anti-parallel β-strands and four α-helices, including an additional short N-terminal helix (α0), which is absent in *Hp*TonB_92_ (Figure 2C). The extra short N-terminal helix (α0) interacts with the last β-strand (β3). The β3 strand coincides with the region where we observed more substantial chemical shift differences between *Hp*TonB_92_ and *Hp*TonB (36–285) (Figures 2C, **5A,C**).

**Table 1:** Experimental data for the NMR structure calculation and the structural statistics of the 20 energy-minimized conformers of *Hp*TonB_107_.

**Quantity**	**Value**
Completeness of resonance assignments (%)^a^	
Backbone	98.9
Side-chain, aliphatic	96.5
Side-chain, aromatic	82.0
NOE upper distance limits	1,851
Short-range NOE (*i* – *j* ≤ 1)	892
Medium-range NOE (1 < *i* – *j* <5)	350
Long-range NOE (*i* – *j* ≥ 5)	609
Residual CYANA target function	0.96 ± 0.03
Residual NOE violation	
Number ≥ 0.2 Å	1
Maximum (Å)	0.16 ± 0.021
Residual dihedral angle violations	
Number ≥ 2.5	0
Amber energies (kcal·mol^−1^)	
Total	−82925.523 ± 3894.06
Van der Waals	13987.97 ± 703.60
Electrostatic	−110727.87 ± 4876.84
rmsd from ideal geometry	
Bond length (Å)	0.0239 ± 0.00007
Bond angles (°)	2.266 ± 0.024
rmsd to mean coordinate^b^^,^^c^	
Backbone (183 – 283) (Å)	0.40 ± 0.05
Heavy atoms (183 – 283) (Å)	0.86 ± 0.06
Ramanchandran plot statistics (%)^c^	
Most favored regions	99.5
Allowed region	0.5
Disallowed region	0
PDB code	6SLY

a *Backbone includes C′, Cα, Cβ, N, and H atoms, except the N-terminal amine. For side chains, excluded are the highly exchangeable groups (Lys amino, Arg guanidino, Ser/Thr/Tyr hydroxyl, His δ1/ϵ2) as well as non-protonated carbons and nitrogens*.

b *As determined by MOLMOL (Koradi et al., 1996)*.

c *Derived from PSVS (MolProbity) (Bhattacharya et al., 2007)*.

### Design for Segmental Isotopic Labeling of *Hp*TonB (36–285)

We believe that the NMR structure of *Hp*TonB_107_ represents the CTD of TonB in the full-length context better than *Hp*TonB_92_ because of the higher similarity of the HSQC spectrum to that of *Hp*TonB (36–285). To examine the structure and dynamics of the CTD, we performed segmental isotopic labeling of *Hp*TonB (36–285) lacking the transmembrane region. The presence of a proline-rich repeating sequence in the LCCR of *Hp*TonB suggests that the LCCR is unstructured compared to the CTD, and likely to have very different dynamical properties due to the increased flexibility (Figure S1). For protein dynamics analysis, series of HSQC spectra are typically recorded with various delay times to measure the longitudinal (T_1_) and transverse (T_2_) relaxation times. Thus, signal overlaps in the HSQC spectra of the uniformly ^15^N-labeled *Hp*TonB (36–285) interfere with the ^15^N relaxation experiments, thereby reducing the reliability of the relaxation data and the number of data points. Therefore, the reduction of the signal degeneracy in the HSQC spectra by segmental isotopic labeling could increase the number of probe residues that could be used for ^15^N relaxation analysis of the CTD in the presence of the LCCR as well as the LCCR itself.

In this work, we chose a split MCM2 intein from *Halorhabdus utahensis (Hut*MCM2) for the production of segmental isotope-labeled samples, instead of the frequently used naturally split DnaE intein from *Nostoc punctiforme (Npu*DnaE intein) (Iwai et al., 2006). Whereas, the robust *Npu*DnaE intein has a Cys residue at the +1 position, the *Hut*MCM2 intein has Ser at the +1 position (Ellilä et al., 2011; Ciragan et al., 2016). *Hut*MCM2 intein thus enables us to make use of a naturally occurring Ser residue in the sequence of *Hp*TonB. Additionally, we decided to perform the protein ligation *in vitro* to prevent isotopic scrambling, which might interfere with the ^15^N relaxation analysis (Züger and Iwai, 2005). We split *Hp*TonB between Lys154 and Ser155 and fused each part with the split *Hut*MCM2 intein fragments. To have the native sequence of *Hp*TonB (36–285), we used Lys154 at the −1 position of the N-terminal split *Hut*MCM2 intein, compromising the splicing efficiency (Ciragan et al., 2016). Thus, the split *Hut*MCM2 intein ligation product will retain the native *Hp*TonB sequence at the junction. Such scarless segmental isotopic labeling approaches have been challenging despite the plethora of available methods.

### Segmental Isotopic Labeling of *Hp*TonB(36–285)

**Figure 3:**
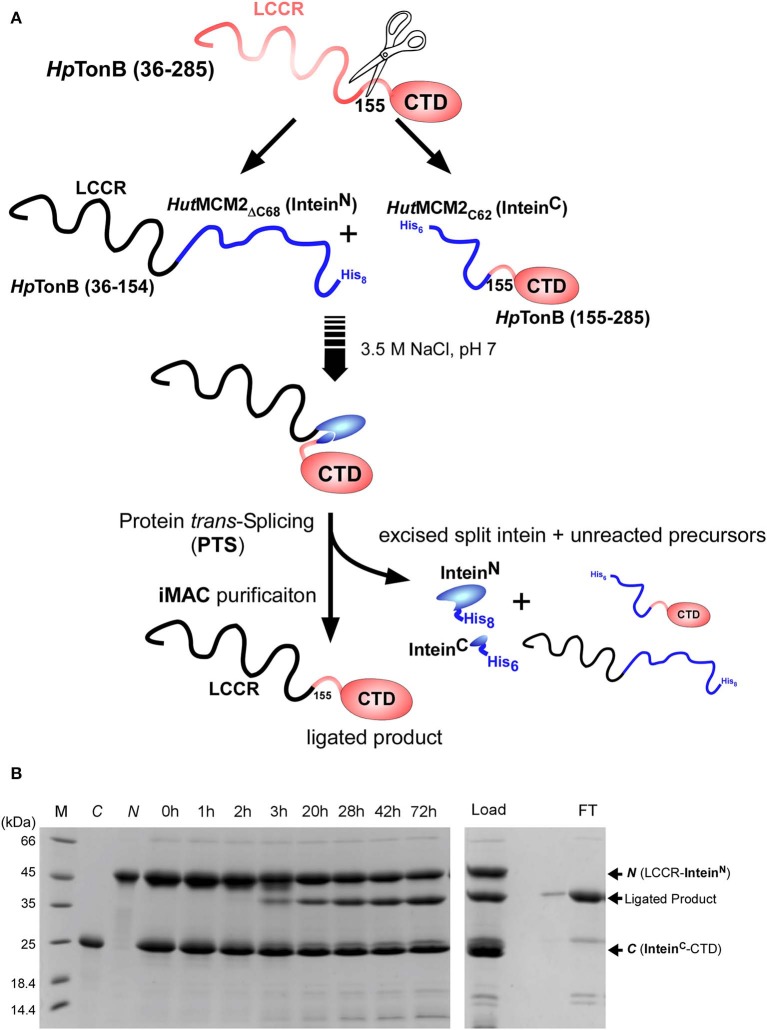
Segmental labeling of *Hp*TonB (36–285). **(A)** Schematic diagram showing the scheme for segmental labeling either the C-terminal or N-terminal domain of *Hp*TonB (36–285) by the split *Hut*MCM2 intein. **(B)** *In-vitro* ligation of segmentally labeled *Hp*TonB (36–285) by PTS using the salt-inducible *Hut*MCM2 intein with Lys at the −1 position. SDS-PAGE analysis shows the time course of the protein ligation reaction. M, *C*, and *N* stand for molecular weight marker, C-terminal and N-terminal precursors before mixing, respectively. Lanes 0–72 h indicate the time (hours) of the ligation reaction after mixing the N- and C-terminal precursors in 3.5 M NaCl. The right panel shows the SDS-PAGE analysis of the IMAC purification step, which removes His-tagged precursors and excised intein fragments simultaneously. “Load” and “FT” indicate the sample loaded on the IMAC column and the flow-through fraction, respectively. *N* and *C* indicate the N- and C-terminal precursors, respectively.

We used a similar strategy for segmental isotopic labeling of *Hp*TonB with two different labeling schemes for this study, as previously reported and depicted in Figure 3A (Ciragan et al., 2016). Briefly, *Hp*TonB (36–154) was fused with the N-terminal split fragment of the *Hut*MCM2 intein carrying a C-terminal octahistidine tag (*Hut*MCM2_Δ*C*62_-H_8_) (Ciragan et al., 2016). We introduced the octahistidine tag instead of a commonly used hexahistidine tag to account for the highly negatively charged *Hut*MCM2 intein reducing the binding to immobilized metal chelate columns. The C-terminal fragment of *Hp*TonB (155–285) was fused to the C-terminal split *Hut*MCM2 intein with an N-terminal hexahistidine tag (H_6_-*Hut*MCM2_C42_). The short names of *Hut*MCM2_Δ*C*62_ and *Hut*MCM2_C42_ for the split fragments are termed for convenience according to the previously reported definition (Aranko et al., 2014b). These two precursor fragments were independently expressed and purified using LB and [20% ^13^C, 100% ^15^N]-containing M9 media. We ligated the two independently purified precursors *in vitro* after mixing at equimolar concentrations with different labeling combinations. To initiate a *trans*-splicing reaction by the salt-inducible split intein, we elevated the NaCl concentration to 3.5 M to reconstitute an active conformation of the *Hut*MCM2 intein either by rapid dilution or dialysis. The reaction mixture under the high salinity was incubated at room temperature for 1–3 days (Figure 3B). Despite the compromised ligation efficiency due to Lys at the −1 position, the histidine tags incorporated into the split *Hut*MCM2 intein fragments facilitated the efficient removal of both unreacted precursors and reacted split intein fragments (Figure 3A). We obtained 5.8 mg and 47.3 mg of [^15^N, 36–154]-labeled *Hp*TonB (36–285) and [^15^N, 155–285]-labeled *Hp*TonB (36–285), respectively.

### Backbone Assignments of *Hp*TonB(36–285) Using Segmentally Isotope-Labeled Samples

**Figure 4:**
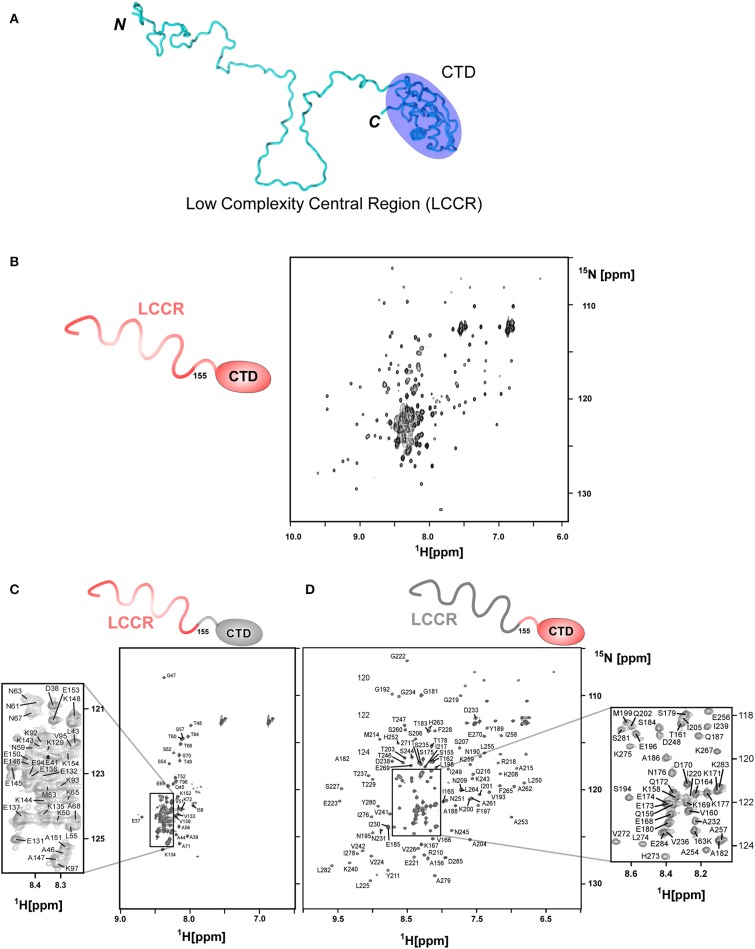
Segmental labeling of *Hp*TonB (36–285) demonstrated by [^1^H, ^15^N]-HSQC spectra. **(A)** A cartoon model of *Hp*TonB (36–285), highlighting the globular CTD and the remaining proline-rich low complexity central region (LCCR). **(B)** [^1^H, ^15^N]-HSQC spectrum of the uniformly ^15^N-labeled *Hp*TonB (36–285). **(C)** [^1^H, ^15^N]-HSQC spectrum of the segmentally ^15^N-labeled LCCR (residues 36–154) of *Hp*TonB (36–285). **(D)** [^1^H, ^15^N]-HSQC spectrum of segmentally ^15^N-labeled CTD (residues 155–285) of *Hp*TonB (36–285).

**Figure 5:**
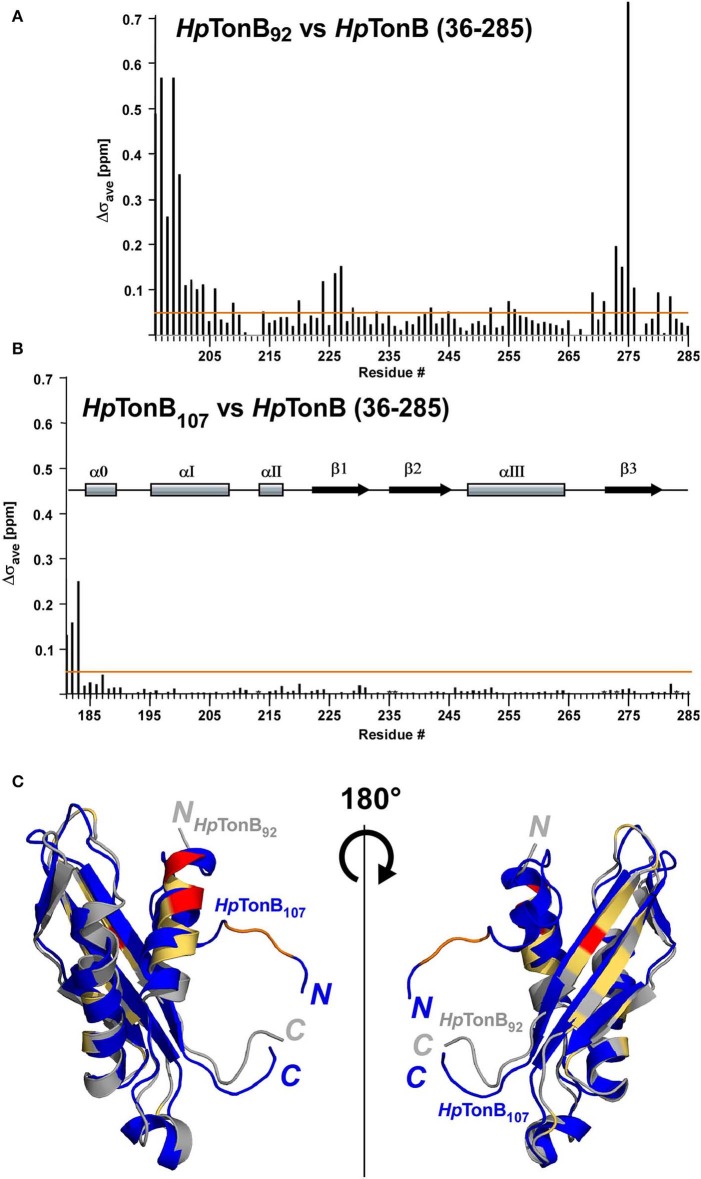
The effects on chemical shifts by dissection of the full-length of *Hp*TonB. **(A)** Chemical shift differences (Δσ_ave_) between *Hp*TonB_92_ and *Hp*TonB (36–285). **(B)** Chemical shift difference (Δσ_ave_) between *Hp*TonB_107_ and *Hp*TonB (36–285). The chemical shift difference (Δσ_ave_) was derived from the equation: Δσ_ave_ = ((ΔδH)^2^ + (0.154 × ΔδN)^2^)^1/2^. Orange lines indicate Δσ_ave_ = 0.05 ppm. The locations of the secondary structural elements of *Hp*TonB_107_ are indicated above the bar graph. **(C)** Ribbon drawings of the superimposition of the two structures of *Hp*TonB_92_ and *Hp*TonB_107_ shown in gray and blue, respectively. Residues with >0.3 ppm for Δσ_ave_ are colored in red. Residues above the threshold of Δσ_av_ = 0.05 ppm are colored in orange for *Hp*TonB_107_ and light orange for *Hp*TonB_92_. The N- and C-termini are indicated by *N* and *C*, respectively.

Segmentally isotope-labeled *Hp*TonB (36–285) samples facilitated more reliable resonance assignments of the crowded regions of the NMR spectrum between 8.2 and 8.5 ppm of the ^1^H dimension, where NMR signals originate mainly from the N-terminal low complexity region (Figure 4). 71.1% of all backbone resonance assignments (H^N^, N^H^, C^α^, C^β^, and C′) of *Hp*TonB (36–285) were completed by making use of the two segmentally labeled samples. Of the backbone amide ^15^N and ^1^H resonances, 78.6% were assigned. Despite relying on segmental isotopic labeling, NMR signals from the proline-rich repeating amino acid sequences in the N-terminal low complexity region were not entirely resolved in the [^1^H,^15^N]-HSQC spectrum (Figure 4C; Figure S1). However, it was possible to unambiguously compare the chemical shifts of the CTD with and without the LCCR. Whereas, the previously reported *Hp*TonB_92_ indicated notable chemical shift changes when compared with *Hp*TonB (36–285) (Figure 5A), the chemical shift difference was much smaller for *Hp*TonB_107_ (Figure 5B). This observation confirms that the NMR structure of *Hp*TonB_107_ likely resembles that of the full-length *Hp*TonB protein. The differences in the chemical shifts of a few N-terminal residues, including Gly181, Ala182, and Thr183, can be attributed to the higher flexibility in the N-terminal end, as observed in the ^15^N relaxation rates (see below).

### Backbone Dynamics of *Hp*TonB (36–285) Investigated by ^15^N Relaxation Times Using Segmentally Isotope-Labeled Samples

^15^N relaxation times have been conveniently used for investigating the internal dynamics of proteins (Palmer, 2004). However, the typical ^15^N relaxation analysis for N-H bonds used for protein dynamics requires quantifications of peak volumes in a series of [^1^H, ^15^N]-HSQC spectra with different relaxation delays. When peaks are not well-resolved in a [^1^H,^15^N]-HSQC spectrum, the quantification of the ^15^N relaxation rate becomes unreliable or even unavailable. Segmental isotopic labeling can alleviate the high signal degeneracy, thereby increasing the number of probes that can be used for NMR analysis. It is thus an attractive method, particularly when no sequence alternation is involved. We combined the ^15^N relaxation analysis of *Hp*TonB (36–285) from the two differently segmentally isotope-labeled samples (one sample was ^15^N-labeled for residues 36–154 of *Hp*TonB, and the other had ^15^N-labeling for residues 155-285) (Figure 4). The segmental isotope-labeled samples indeed increased the number of probes used for the ^15^N relaxation analysis, thereby enhancing the reliability of the relaxation analysis.

**Figure 6:**
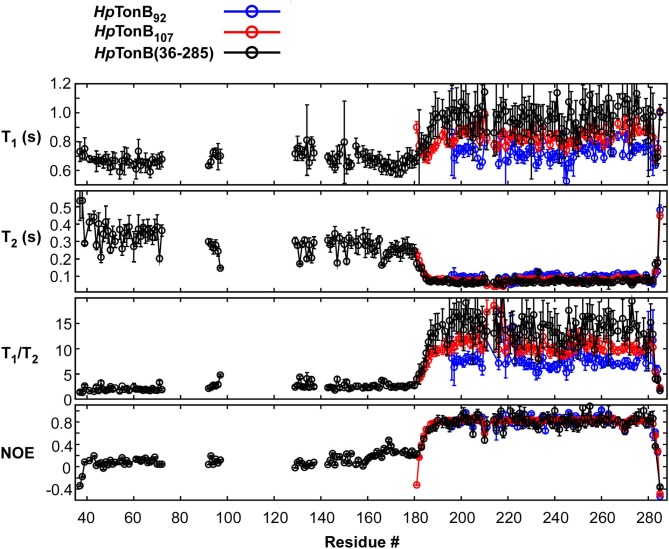
^15^N relaxation data obtained for *Hp*TonB_92_, *Hp*TonB_107_, and *Hp*TonB (36–285) are shown in blue, red, and black, respectively. The longitudinal relaxation time (T_1_), transverse relaxation time (T_2_), T_1_/T_2_ ratios, and heteronuclear ^15^N{^1^H}-NOE are plotted against the residue number. Residues with overlaps and residues with ambiguous assignments were removed from the plots.

Here, we compared the ^15^N relaxation rates from three different constructs of *Hp*TonB_92_, *Hp*TonB_107_, and *Hp*TonB (36–285) by making use of segmentally labeled samples (Figure 6). The ^15^N relaxation studies showed that the structured CTD has distinct protein dynamics from the pro-rich LCCR, which has fast dynamics in the picosecond-to-nanosecond time scale as observed from the reduced heteronuclear NOEs (Figure 6). On the other hand, the differences in the spin relaxation times for the different CTDs are small. Assuming that T_1_ and T_2_ are independent of internal motions and that the rotation of CTD is spherically isotropic (Kay et al., 1989), the overall correlation times estimated from the T_1_/T_2_ ratio of the globular regions were 5.8, 6.9, and 8.2 ns for *Hp*TonB_92_, *Hp*TonB_107_, and *Hp*TonB (36–285), respectively (Ollila et al., 2018). Since even small anisotropy could affect the analysis of protein dynamics (Lüginbuhl et al., 1997), the detailed analysis of rotational dynamics taking into account the effect of anisotropy and disordered linker using molecular dynamics simulation is currently under investigation and will be published elsewhere. The overall correlation times for the truncated constructs are only slightly shorter than *Hp*TonB (36–285), even though the molecular size of *Hp*TonB (36–285) is double of *Hp*TonB_107_. Because the NMR structure of *Hp*TonB_107_ likely represents the structure in the full-length context, we assume that the dynamical properties of the CTD are mainly independent of the very flexible pro-rich LCCR region. The negative heteronuclear NOE values for the N- and C-termini of *Hp*TonB_107_ and *Hp*TonB (36–285) are indicative of rapid local motions. The N-terminal region (181–182) of *Hp*TonB_107_ showed decreased heteronuclear NOEs compared with the corresponding region of *Hp*TonB (36–285). This observation suggests that the truncation of the LCCR in *Hp*TonB_107_ induced flexibility for this region, which is more restricted in *Hp*TonB (36–285). The change in the local dynamics could account for the observed chemical shift differences between *Hp*TonB_107_ and *Hp*TonB (36–285) around this region (Figure 5B).

## Discussion

It has been a common practice in structural biology to dissect a protein into a globular domain for structural investigation by removing flexible or disordered regions because it often facilitates crystallization and also alleviates the signal overlap problem for NMR analysis. However, IDRs and IDPs are increasingly recognized for their functional importance. Thus, structural investigations of proteins in their full-length contexts, including disordered regions, have become imperative to understand how proteins function in living systems, imposing technical challenges. NMR spectroscopy has the advantage of investigating protein structures with disordered regions because it does not require any crystallization and could be studied under various solution conditions. However, NMR signal assignments could be even more time-consuming and/or difficult when proteins contain repetitive sequences because of the increased complexity and high degeneracy of NMR signals.

Here, we demonstrated scarless segmental isotopic labeling by protein *trans*-splicing for the structural investigation of *Hp*TonB, resulting in no mutation at the ligation site despite the low ligation efficiency. Segmental labeling of *Hp*TonB indeed alleviated the high degeneracy of NMR spectra originating from the low complexity central region, thereby enabling the detailed comparison of the differently dissected CTDs and a full-length construct without TM region. The comparison between the two structures of *Hp*TonB_92_ and *Hp*TonB_107_ demonstrated that a dissected domain does not necessarily represent the structure in the full-length context, which is supported by the more substantial chemical shift differences observed for *Hp*TonB_92_. In line with this observation, we found the instability of *Hp*TonB_92_ because it precipitated during extended NMR measurements.

In summary, our study using *Hp*TonB system highlights the importance of performing structural investigations with the full-length protein or at least fragments close to the full-length. The ^15^N-relaxation analysis by segmentally isotopically labeled sample confirmed that the LCCR of *Hp*TonB (36–285) is flexible with largely independent motions of the CTD. Even though the sequence conservation of LCCR is very weak within the N-terminal and the central regions, the Pro-Lys and Pro-Glu repeats in the LCCR constitute the flexible region, influencing the structural and rotational dynamics of the CTD. It remains elusive how the flexible disordered linker of the LCCR could mediate the biological function in the TonB-dependent transduction system. It would be of remaining interests to investigate the structure and dynamics of TonB inserted into the membrane environment, which might require three-fragment ligation with an additional ligation step. With further improvement of protein ligation by PTS, scarless segmental isotopic labeling by two- and three-fragment ligation is expected to pave the way for NMR studies of unfolded proteins and multi-domain proteins with mixed dynamics as exemplified with *Hp*TonB.

## Materials and Methods

### Production and Purification of Labeled and Unlabeled Precursors

*E. coli* strain T7 Express (New England Biolabs) was transformed with the plasmid encoding either the N-terminal precursor encoding *Hp*TonB (36–154)-*Hut*MCM2_Δ*C*62_-H_8_ (pACRSF5) (Ciragan et al., 2016) or the C-terminal precursor H_6_-*Hut*MCM2_C42_-*Hp*TonB (155–285) (pBHRSF165) (Ciragan et al., 2016) and grown in 50 mL LB medium supplemented with 25 μg/mL kanamycin at 30°C overnight. The pre-cultures were used to inoculate 1 liter for pACRSF5 or 2 liters for pBHRSF165 of LB medium supplemented with 25 μg/mL kanamycin and incubated at 37°C while shaking at 200 rpm. When OD_600_ reached 0.6, expression was induced with a final concentration of 1 mM isopropyl-β-D-thiogalactopyranoside (IPTG), and induction was continued for 4 h.

For the production of the labeled N-terminal precursor protein, 5 mL of pre-culture containing plasmid pACRSF5 was used to inoculate 50 mL of [20% ^13^C, 100% ^15^N]-containing M9 medium with ^15^NH_4_Cl (1 g/L) and a mixture of ^13^C_6_ D-glucose (0.2 g/L) and unlabeled D-glucose (0.8 g/L) as sole nitrogen and carbon sources, respectively, supplemented with 25 μg/mL kanamycin. The culture was incubated at 30°C with shaking at 200 rpm overnight and then transferred into pre-warmed 1950 mL of [20% ^13^C, 100% ^15^N]-containing M9 medium supplemented with 25 μg/mL kanamycin and grown at 37°C under shaking. The protein was induced with a final concentration of 1 mM IPTG when OD_600_ reached 0.6 and lasted 4–5 h.

Expression cultures for the production of labeled C-terminal precursor protein containing plasmid pBHRSF165 were first grown in 2 L of LB medium until OD_600_ reached 0.6. The cultures were then harvested by centrifugation at 1,600 *g* for 12 min at 20°C and gently resuspended in 12 mL of [20% ^13^C, 100% ^15^N]-containing M9 medium. The cell suspension was added to 1.8 L of complete M9 medium containing 25 μg/mL kanamycin and grown at 30°C while shaking at 200 rpm for 4 h. After 20 min, expression was induced with 1 mM IPTG. Cells were harvested by centrifugation at 4,000 *g*, 4°C for 10 min, resuspended in 20 mL of 50 mM sodium phosphate buffer, pH 8.0, 300 mM NaCl, frozen in liquid nitrogen, and stored for further use at −74°C. The cell pellets were thawed and lyzed at 15,000 psi for 10 min, 4°C using an Emulsiflex C3 homogenizer (Avestin). Cell debris was removed by centrifugation at 38,000 *g*, 4°C for 60 min, the supernatant was passed through a 0.45 μm filter and followed by loading on a 5 mL HisTrap HP column (GE Healthcare Life Sciences), which was pre-equilibrated with 50 mM sodium phosphate buffer, pH 8.0, 300 mM NaCl, 10 mM imidazole. After washing with 50 mM sodium phosphate buffer, pH 8.0, 300 mM NaCl, 30 mM imidazole, the protein was eluted with a linear gradient of 30–250 mM imidazole in 50 mM sodium phosphate buffer, pH 8.0, 300 mM NaCl. The elution was dialyzed overnight against phosphate-buffered saline (PBS) at 8°C. N- and C-terminal precursors for segmental labeling were concentrated using ultra-centrifugal devices and frozen in liquid nitrogen for storage at −74°C.

### Salt-Induced Protein Ligation by Protein Trans-Splicing (PTS)

Ligation of *Hp*TonB(36–154)-*Hut*MCM2_Δ*C*62_-H_8_ with H_6_-*Hut*MCM2_C42_-*Hp*TonB (155–285) was performed according to the protocol published previously with slight modifications (Ciragan et al., 2016). Purified ^15^N-labeled C-terminal precursor and the unlabeled N-terminal precursor were mixed in an equimolar ratio at a final concentration of 0.25 mM each, in a total volume of 11 mL with 0.5 M sodium phosphate buffer, pH 7.0, 3.5 M NaCl and 0.5 mM tris(2-carboxyethyl)phosphine hydrochloride (TCEP) and incubated at room temperature for 3 days. The ligation mixture was dialyzed at room temperature against PBS and then against 50 mM sodium phosphate buffer, pH 8.0, 350 mM NaCl. The sample was further diluted twice with fresh buffer before loading onto a 5 mL HisTrap HP column to remove the unreacted precursors and excised intein fragments. For producing *Hp*TonB (36–285) using a ^15^N-labeled N-terminal precursor instead, the two precursor proteins were mixed in an equimolar ratio at 0.325 mM each, in 4 mL PBS and dialyzed overnight at room temperature against 350 mM sodium phosphate buffer, pH 7.0, 3.5 M NaCl, 0.5 mM TCEP. After 24 h, the dialysis buffer was exchanged with PBS for 2 h. The sample was diluted 5-fold with 50 mM sodium phosphate buffer, pH 8.0, 300 mM NaCl, and purified using a 5 mL HisTrap HP column as described above. The flow-through fractions containing ligated segmentally labeled *Hp*TonB (36–285) protein were dialyzed overnight at 8°C against 20 mM sodium phosphate buffer, pH 6.0 for NMR measurements.

### Molecular Cloning and Production of Uniformly Labeled *Hp*TonB_107_

The coding sequence of the 107-residue C-terminal domain of *Hp*TonB (*Hp*TonB_107_) was PCR-amplified from *Helicobacter pylori* genomic DNA using the oligonucleotides I472: TTAAAGCTTAGTCTTCTTTCAAGCTATAAGCGATAG and I812: AAAGGATCCGAGGGGGCCACTTCCGAAGCTCAGGCTTATAACC. The PCR product was digested with *Bam*HI and *Hin*dIII and ligated into pHYRSF53 (Addgene #64696), resulting in pBHRSF159, encoding *Hp*TonB with N-terminal hexahistidine tag and SUMO fusion (H_6_-SUMO-*Hp*TonB_107_) (Guerrero et al., 2015). For protein production, the plasmid was transformed into *E. coli* T7 Express cells (New England Biolabs). The cells were grown overnight at 30°C in 50 ml LB medium supplemented with 25 μg/ml kanamycin. The cells were spun down at 900 *g* for 15 min and gently resuspended in 2 L pre-warmed M9 medium with 25 μg/ml kanamycin, containing ^15^NH_4_Cl and ^13^C-glucose as the sole nitrogen and carbon source, respectively. Further protein expression and purification were performed as described for the N-terminal labeled *Hp*TonB (36–285) precursor, except that the expression culture was grown at 30°C for 5 h. Cells were harvested, lyzed, and purified, as described above. The N-terminal H_6_-SUMO fusion was removed using the SUMO-specific protease Ulp1 and the final [100% ^13^C, 100% ^15^N]-labeled *Hp*TonB_107_ protein was recovered in the flow-through after passage through a 5 mL HisTrap HP column as previously described (Guerrero et al., 2015). The purified protein was dialyzed against 20 mM sodium phosphate buffer, pH 6.0 for NMR analysis, and concentrated using ultrafiltration.

### Sample Preparation for NMR Measurements

Segmentally labeled *Hp*TonB (36–285) or uniformly labeled *Hp*TonB_107_ protein samples in 20 mM sodium phosphate buffer, pH 6.0 were mixed at the given concentrations with the following additives: *Hp*TonB (36–285) with N- or C-terminal segmental labeling, 300 μM, 150 mM NaCl, 5% D_2_O in 220 μL; uniformly labeled *Hp*TonB_107_, 450–500 μM in 250 μL with 10% D_2_O.

### Determination of NMR Solution Structures

For the sequential resonance assignment, a standard set of double and triple resonance spectra was recorded: [^1^H, ^15^N]-HSQC, [^1^H, ^13^C]-HSQC, HNCO, HNCA, HNCACB, HN(CO)CA, HN(CA)CO, CBCA(CO)NH, HCCH-COSY, H(CCCO)NH, (H)CC(CO)NH (Sattler et al., 1999). NMR measurements were performed on Bruker Avance IIIHD 850 MHz spectrometer equipped with a cryogenically cooled probe head at 303 K at the Institute of Biotechnology, University of Helsinki. Resonance assignment was done using CcpNmr Analysis (Vranken et al., 2005). Automated NMR structure calculation was performed by CYANA 3.0, which is based on automated NOESY cross-peaks assignment using previously determined chemical shift values (Güntert et al., 1997; Herrmann et al., 2002). For the structure calculation, triple resonance ^15^N-edited NOESY-HSQC and ^13^C-edited NOESY-HSQC spectra with mixing times of 70 and 90 ms, respectively, were used for *Hp*TonB_107_. The chemical shift values from the sequential resonance assignment were used together with the NOE peak lists for the structure calculation (Güntert et al., 1997; Herrmann et al., 2002). Energy minimization of the structure was done using the 20 best conformers based on the lowest values of the CYANA target function by AMBER 14 (Cornell et al., 1995; Pearlman et al., 1995). Quality analysis of the structure was performed using PSVS 1.5 validation software suite (Bhattacharya et al., 2007), PROCHECK-NMR (Laskowski et al., 1996), and WHAT IF (Vriend, 1990).

### Backbone Dynamics of *Hp*TonB

The spin relaxation times of *Hp*TonB_92_ were published previously (Ollila et al., 2018), and *Hp*TonB_107_ relaxation times were measured with the identical setup described in that publication. For *Hp*TonB (36–285) with residues 36–154 or 155–285 labeled, the same pulse sequence (Kay et al., 1989; Barbato et al., 1992; Farrow et al., 1994) was used with slightly modified delay times. In *Hp*TonB (36–285) experiments with residues 36–154 labeled, the delay times of 50, 100, 200, 300, 500, 700, and 900 ms were used for T_1_, and delay times of 51, 68, 85, 119, 153, 187, 220, and 254 ms were used for T_2_. The recycling delay of 3.5 s was used for both T_1_ and T_2_, and 5.0 s for heteronuclear NOE. The same settings were used for the segmentally [^15^N, 154–285]-labeled sample of the *Hp*TonB (36–285), except for the T_2_ delays, which were 51, 68, 85, 102, 136, 170, 204, and 237 ms, and three-second recycling delays for T_1_ and T_2_. The temperature in these experiments was 298 K. The T_1_ and T_2_ relaxation data were processed and analyzed using Bruker Dynamic Center software (version 2.5.5) and the NOE relaxation times were processed and analyzed using CcpNmr Analysis software (version 2.4.2) (Vranken et al., 2005). The approximate time-scales for the CTD rotation were estimated, minimizing the equation (11) from Ollila et al. (2018) with respect to the experimentally measured T_1_/T_2_ ratios as originally proposed by Kay et al. (1989).

## Data Availability Statement

The coordinates and the chemical shifts for this study can be found in the protein data bank (http://www.rcsb.org/pdb/home/home.do) with PDB ID code: 6SLY and in BMRB (http://www.bmrb.wisc.edu/) with the accession number: 34425.

## Author Contributions

HI and OS designed and supervised the project. AC, HB, and KM have contributed to the production of NMR samples. AC and SB performed the NMR analysis and structure determination. OS contributed to the relaxation studies. All authors contributed to writing of the manuscript.

### Conflict of Interest

The authors declare that the research was conducted in the absence of any commercial or financial relationships that could be construed as a potential conflict of interest.

## Supplementary Material

Figure S1 Sequence alignment of TonB protein from four gram-negative bacteria (HP, *Helicobacter pylori*; EC, *Escherichia coli*; ST, *Salmonella typhimurium*; PA, *Pseudomonas aeruginosa*). Black color indicates identical residues, and gray color indicates residues with similar chemical characteristics. The asterisk (^*^) indicates the position of the conserved histidine (except for *HP*) in the transmembrane domain. The domain arrangement of *Hp*TonB is shown above the amino-acid sequence.

Figure S2 Comparison of the two [^1^H, ^15^N]-HSQC spectra of **(A)** *Hp*TonB_92_ and **(B)** *Hp*TonB_107_ Sequence-specific assignments are marked for each peak with one-letter codes for amino-acid types.

## Abbreviations

CTD: C-terminal domain
DTT: dithiothreitol
EPL: expressed protein ligation
GB1: B1 domain of IgG binding protein G
HSQC: Heteronuclear Single Quantum Coherence
*Hp*: *Helicobacter pylori*
*Hut*: *Halorhabdus utahensis*
IDP: intrinsically disordered protein
IDR: intrinsically disordered protein region
IMAC: immobilized metal affinity chromatography
IPTG: isopropyl β-d-1-thiogalactopyranoside
LCCR: low complexity central region
PMF: proton motive force
NMR: nuclear magnetic resonance
*Npu*: *Nostoc punctiforme*
OM: outer membrane
PBS: phosphate-buffered saline
PTS: protein *trans*-splicing
TCEP: tris(2-carboxyethyl)phosphine
TBDT: TonB-dependent transporter
TM: *trans*membrane.
