# Supplementary material for: NMR Structure and Dynamics of TonB Investigated by Scar-Less Segmental Isotopic Labeling Using a Salt-Inducible Split Intein
Source: Front Chem. 2020 Mar 19;8:136. doi: 10.3389/fchem.2020.00136 (PMC7098700; doi:10.3389/fchem.2020.00136)
Supplement: Figure S1 — Sequence alignment of TonB protein from four gram-negative bacteria (HP, Helicobacter pylori; EC, Escherichia coli; ST, Salmonella typhimurium; PA, Pseudomonas aeruginosa). Black color indicates identical residues, and gray color indicates residues with similar chemical characteristics. The asterisk (*) indicates the position of the conserved histidine (except for HP) in the transmembrane domain. The domain arrangement of HpTonB is shown above the amino-acid sequence. [file Data_Sheet_1.pdf]

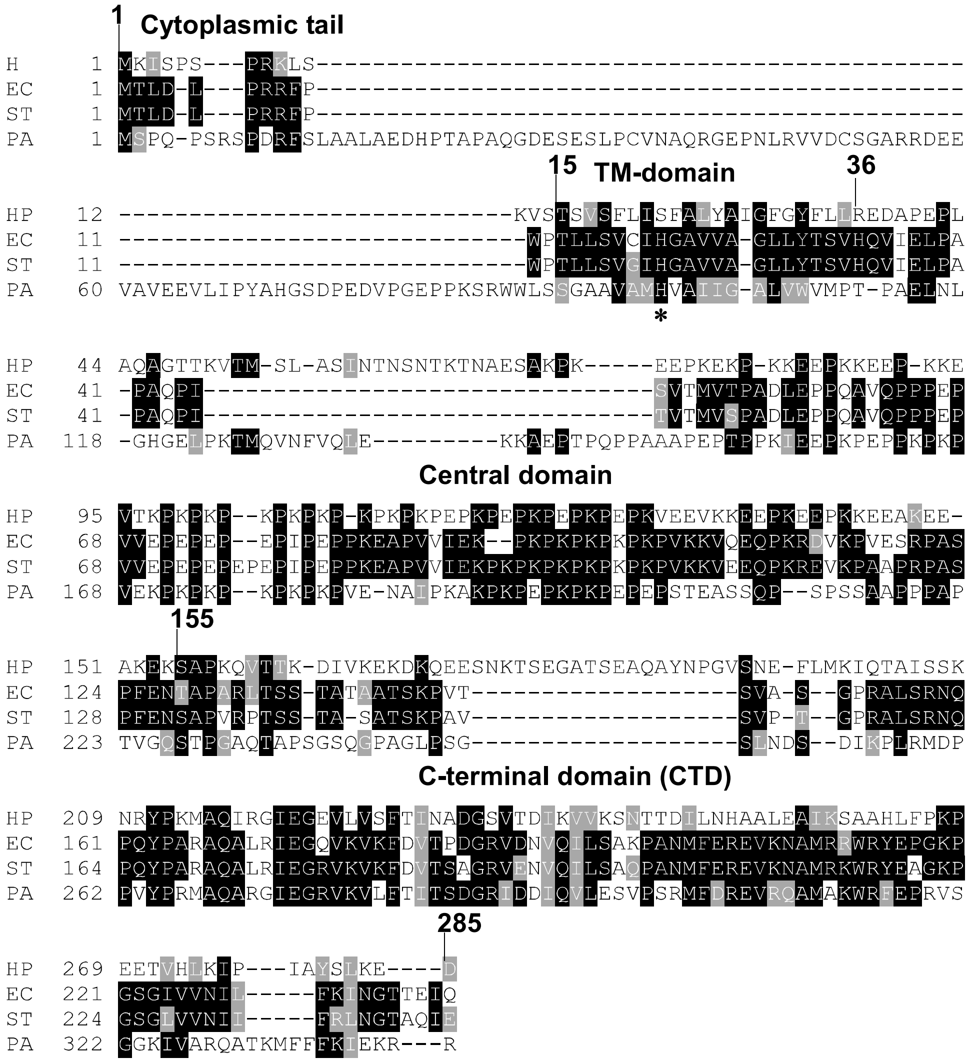

Supplemental Fig. S1

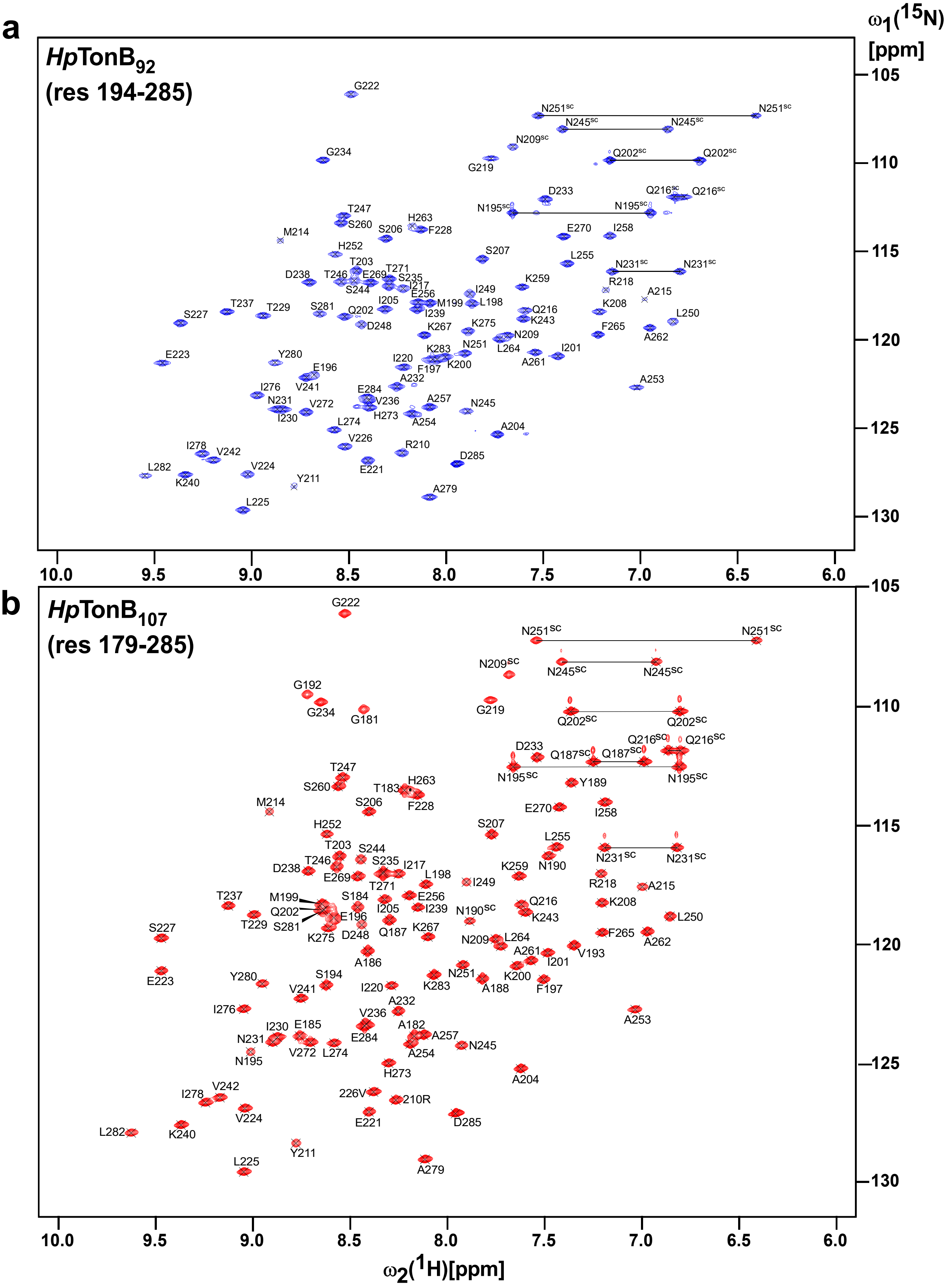

Supplemental Fig. S2
